# Prevalence of Signs and Symptoms of Temporomandibular Disorders and Their Association with Emotional Factors and Waking-State Oral Behaviors on University Students: A Cross-Sectional Study

**DOI:** 10.3390/healthcare13121414

**Published:** 2025-06-12

**Authors:** Davide Cannatà, Marzio Galdi, Mario Caggiano, Alfonso Acerra, Massimo Amato, Stefano Martina

**Affiliations:** Department of Medicine, Surgery and Dentistry “Scuola Medica Salernitana”, University of Salerno, Via Allende, 84081 Salerno, Italy

**Keywords:** temporomandibular disorders, TMD, anxiety, depression, stress, epidemiology

## Abstract

**Background/Objective:** This cross-sectional study assessed the prevalence of temporomandibular disorders (TMD) among Italian university students and their association with emotional factors and parafunctional behaviors. **Methods:** A total of 321 students participated in this study. TMD signs and symptoms were evaluated using the DC/TMD criteria through clinical examinations and self-report questionnaires: physical (Symptom Questionnaire), psycho-emotional (PHQ-9, PHQ-15, PHQ-4, and GAD-7), and wake-state oral behaviors (Oral Behavior Checklist, OBC). The Mann–Whitney U test assessed associations between TMD, sociodemographic data, oral behaviors, and psychological vulnerability (*p* < 0.05). **Results:** Pain-related symptoms were present in 37% of students (male/female ratio 1:2.7; *p* < 0.001), and joint dysfunction in 28%, with no gender differences. The median score of PHQ-9 (2.0; interquartile range IQR 5.0), PHQ-15 (2.0; IQR 5.0), PHQ-4 (3.0; IQR 6.0), and GAD-7 (3.00; IQR 6.0) suggested negligible severity of anxious mood, depressed mood, and somatic symptoms among the university students. However, all scores were noticeably higher in students with pain-related TMD compared to pain-free ones (*p* < 0.05). OBC scores were significantly related to PHQ (*p* < 0.001), GAD-7 (*p* < 0.001), and pain symptoms (*p* < 0.001). Science faculty students (S) showed higher OBC scores than humanities ones (H; S: 20.0; IQR 13.0 vs. H: 16.0; IQR 14.0; *p* < 0.001), and an H/S ratio of 1:2 was found in pain prevalence (*p* < 0.05). **Conclusions:** This study found a high prevalence of TMD signs and symptoms, particularly pain-related, among university students, strongly linked to emotional factors and oral behaviors.

## 1. Introduction

Temporomandibular disorders (TMD) is an umbrella term that embraces a group of clinical conditions involving the temporomandibular joints (TMJ), the masticatory muscles, or both [1]. These conditions are characterized by pain-related symptoms, such as articular or muscular pain, and/or joint dysfunctional signs, such as alteration of the physiological jaw dynamics or TMJ noises (clicking or crepitus) [2].

TMD arises from a multifactorial interplay of several determinants acting on a biological predisposition, involving structural abnormalities, muscle dysfunction, ligamentous laxity, hormonal and metabolic changes, systemic diseases, and genetic mutations [3]. Determinants in the onset and/or persistence of TMD have been recognized in some forms of injury or trauma [4], including micro-trauma derived from oral behaviors (OBs), a broad range of activities related to the jaws, which can occur during either sleep or wakefulness [5,6]. OBs can be divided into three groups: sleeping-state oral activities, including sleep bruxism (i.e., phasic or tonic masticatory muscle activities occurring during sleep) [7] and maintaining sleep postures that apply pressure to the jaw area; waking-state functional oral activities, including physiological oral functions, such as mastication, communication, swallowing, and breathing; and waking-state non-functional activities, including awake bruxism (i.e., masticatory muscle activities characterized repetitive or sustained tooth contact and/or by the bracing or thrusting of the mandible while awake) [7] and other holding activities [8].

Among OBs, bruxism (both sleep and awake) has been the most studied in the TMD etiopathogenesis, since the global prevalence of its co-occurrence with TMD is 17% [9]. However, the relationship between bruxism and TMD remains ambiguous. The obvious hypothesis that the presence of OBs overuses the masticatory system, leading to the occurrence or worsening of the TMD symptoms, finds weak evidence in literature [10]. Moreover, stress, psychological symptoms, and negative mood that have been associated with both OBs [5] and TMD [11] may act as confounding factors linking OBs and TMDs. Indeed, on the one hand, the limbic system, which is central to emotional processing, along with the involvement of gamma loop pathways connected to the masticatory muscles, may contribute to heightened emotional tension manifesting as increased contractile activity in these muscles, resulting in bruxism [12,13]. On the other hand, psychological factors, such as individual, interpersonal, and situational variables, can reduce the individual’s capability to function adaptively, increasing pain sensitivity, reducing perception and tolerance of physical symptoms, and thus resulting in TMD rising or chronicization [11,12].

Nociceptive signals are processed in different brain areas including the somatosensory cortex, anterior cingulate cortex, insula, amygdala, thalamus, and hypothalamus. Neural pathways originating from the insular and prefrontal regions project through the periaqueductal gray (PAG) to various brainstem centers and ultimately reach the spinal cord, playing a role in top-down regulation of pain signals. This regulatory mechanism can exert both facilitative and suppressive effects and engages among other substances, including endogenous opioids as well as neurotransmitters like norepinephrine and serotonin [14]. Anxiety, depression, and emotional distress are associated with altered levels of neurotransmitters, receptor expression, molecular pathways, and structural alterations in the brain areas mentioned above, modulating pain experience, including muscle pain [15,16]. Coherently, a significant proportion of TMD patients have a history of stress-related disorders and major life events [17], and patients with TMD frequently exhibit elevated levels of anxiety relative to healthy individuals [18]. Furthermore, patient’s concentration on pain may exacerbate its perception [19].

A part of the population most susceptible to having psychosocial disorders was university students, because both of their young age and academic workload, which is very high, with tight deadlines, demanding exams, and high pressure to perform well. Worldwide, it has been estimated that 33% experience at least one or more signs and/or symptoms of a mental sphere disorder, such as anxiety, depression, or catastrophizing [20]. The mental health of university students has become a topic of increasing interest, with a significant focus on the rising rates of stress and anxiety within this group. Several studies have drawn attention to this worrying trend [21,22,23]. In particular, a global survey conducted by the World Health Organization (WHO) revealed the widespread prevalence of generalized anxiety among university students worldwide [24]. Given the profound impact of anxiety on students’ social interactions, academic performance, and overall well-being, it was crucial to identify its risk factors and understand the underlying mechanisms to develop effective intervention strategies [25].

Considering the high prevalence of stress, anxiety, and depressed mood among university students, and their co-occurrence with TMD, several investigation in different countries, including Jordan, Brazil, and Saudi Arabia, have assessed the prevalence of TMD signs and symptoms in local university students, finding higher values than in the general population [26,27,28].

In Italy, the prevalence of TMD disorders, assessed through a reproducible and scientifically validated tool, i.e., the Diagnostic Criteria for Temporomandibular Disorders (DC/TMD) by Schiffman et al. [29], in a general adult population has been carried out [1]. Instead, no data on the prevalence of TMD signs and symptoms among university students in Italy are available. Therefore, the primary objective of this study was to determine the prevalence of TMD among Italian university students. Furthermore, this study investigated the association of TMD signs and symptoms with emotional factors and OBs, in order to identify risk groups for the development of TMD.

## 2. Materials and Methods

### 2.1. Study Participants

In the present cross-sectional study, the sample was selected from 1465 consecutive patients referred to the Dental Complex Operating Unit of Dentistry of the Hospital “San Giovanni di Dio e Ruggi d’Aragona” in Salerno, for a dental examination, between December 2024 and March 2025. This study was approved by the Campania 2 Ethics Committee (No. 2024/30960). Each patient underwent an exhaustive clinical examination, including collection of detailed medical and dental history, intraoral and extraoral inspection, periodontal probing, dental percussion (if needed), pulp sensibility and vitality tests (if needed), and palpation of lymph nodes of the head and neck. Additionally, panoramic radiographs of each patient were requested and analyzed. Written informed consent for the objective examination and the processing of sensitive data was obtained from each patient before the visit began.

Patients who were considered eligible for participation in this study were those who were undergraduate students registered in a degree program at the University of Salerno, with no medical or psychological condition hindering their comprehension or questionnaire completion, in which no cause of orofacial pain other than muscle and TMJ pain could be identified. The inclusion and exclusion criteria are summarized in Table 1.

Eligible subjects were identified, and each of them received a comprehensive explanation of the objective of this study and methods of data collection and procession and was asked to give written consent to participate in this study.

Eligible subjects who consented to participate in this study underwent a physical assessment of muscle and TMJ pain, a psychological assessment of somatization, distress, anxiety, and depression, and an assessment of waking-state OBs.

### 2.2. Physical Assessment

The DC/TMD Axis I assessment instruments were used to detect any physical signs or symptoms of TMD, including the Symptom Questionnaire and the Clinical Examination Form, using Italian translation [29]. Moreover, patients’ sociodemographic characteristics were gathered, including sex, age, and area of this study (humanistic or scientific).

The clinical examination was independently performed by two examiners (D.C. and M.G.), who were previously instructed in taking measurements of jaw dynamics, muscle and joint palpation, and joint auscultation. Inter-examiner reliability in the measurement process was assessed using Cohen’s kappa coefficient [30]. For jaw movements, the mean of the measurements of the two examiners was considered. For joint palpation and auscultation, a third clinician (S.M.) was considered in case of disagreement between the examiners.

The same examiners made the diagnoses following the Diagnostic Criteria. Accordingly, subjects could be affected by pain disorders, including myofascial pain, arthralgia, headache attributed to TMD, in which pain is the main clinical symptom; or joint disorders, including disc displacement with reduction, disc displacement with intermittent locking, disc displacement without reduction with limited opening, disc displacement without reduction without limited opening, degenerative joint disease, subluxation/luxation, in which alterations in jaw dynamics represent the main clinical signs.

### 2.3. Psychological Assessment

Individuals’ psycho-emotional profiles were evaluated using the Italian versions of the DC/TMD Axis II assessment instruments [31], including the Italian version of the Generalized Anxiety Disorder Questionnaire-7 (GAD-7) for scoring the anxious mood and behavior [32,33], the Italian version of the Patient Health Questionnaire-9 (PHQ-9) for screening depressed mood [34,35], the Italian version of the Patient Health Questionnaire-4 (PHQ-4) for assessing psychological distress [36], and the Italian version of the Patient Health Questionnaire-15 (PHQ-15) for detecting non-specific physical symptoms, also referred as functional symptoms or medically unexplained symptoms [37,38]. All questionnaires were retrieved from the International Network for Orofacial Pain and Related Disorders Methodology (INfORM) website (https://inform-iadr.com/index.php/tmd-assessmentdiagnosis/dc-tmd-translations/, accessed on 7 November 2024).

Accordingly, anxious mood was considered negligible, mild, moderate, or severe when GAD-7 score was 0 to 4, 5 to 9, 10 to 14, 15 to 21, respectively; depressed mood was considered negligible, mild, moderate, moderate-severe, and severe when PHQ-9 score was 0 to 4, 5 to 9, 10 to 14, 15 to 19, and 20 to 27, respectively; psychological distress was rated as absent, mild, moderate, or severe, when PHQ-4 scored between 0 and 2, 3 and 5, 6 and 8, and 9 and 12, respectively; and severity of somatic symptoms was considered negligible, low, medium, or high, when PHQ-15 scored between 0 and 4, 5 and 9, 10 and 14, and 15 and 30, respectively. The cut-off scores were chosen based on previous studies [37,39,40,41].

Other assessment instruments of DC/TMD Axis II, such as the pain drawing, the Graded Chronic Pain Scale (GCPS) version 2, and the Jaw Functional Limitation Scale-20 (JFLS-20), were administered to subjects but were not considered for the purpose of this study as these instruments assess aspects not relevant to the objectives of this study, namely the severity and impact of chronic pain (GCPS) [42], or information already came through the clinical examination, such as facial pain location (pain drawing) [42], and functional limitation of the jaws (JFLS-20) [42].

### 2.4. Assessment of Waking-State OBs

OBs were assessed using the Italian version of the Oral Behavior Checklist (OBC) [43], a self-reported 21-item questionnaire evaluating OBs performed during the preceding month [44]. The Italian translation was retrieved from the International Network for Orofacial Pain and Related Disorders Methodology (INfORM) website (https://inform-iadr.com/index.php/tmd-assessmentdiagnosis/dc-tmd-translations/, accessed on 7 November 2024). For the purposes of this study, the first two questions of OBC, assessing sleep-related behaviors, were excluded.

Accordingly, the presence of parafunctional behaviors was considered low when the OBC summary score ranged between 0 and 16, and high when the summary score ranged between 17 and 76 [45].

### 2.5. Sample Size Calculation

The target sample size was estimated using R Software, version 4.1.0 (May 2021; R Foundation for Statistical Computing, Vienna, Austria), based on Cochran’s sample size formula for prevalence studies [46].

Since the estimated prevalence of TMD in general population is about 34% [47] and a total number of students at the University of Salerno is about 40,000 according to web.unisa.it extracted on May 2024, the required sample size was 320 for the margin of error of 0.5% in estimating the prevalence with 95% confidence.

### 2.6. Statistical Analysis

Data obtained from the questionnaires and clinical examination were summarized using descriptive statistical analysis and mean and standard deviations were computed for quantitative data, whereas frequencies and percentages were calculated for qualitative data. The Shapiro–Wilk test was used to check whether data set has a normal distribution and, consequently, to choose the appropriate statistical test to analyze them according to their distribution.

The chi-squared test was used to assess the association between sociodemographic data and the presence of TMD signs and symptoms.

The Mann–Whitney U test was performed to assess the differences between the subjects with and without signs and symptoms of TMD in GAD-7, PHQ-9, PHQ-15, PHQ-4, and OBC scores.

The Spearman rank correlation coefficient was calculated to assess the possible association between OBC and PHQ-9, PHQ-15, PHQ-4, and GAD-7 scores. All tests were deemed significant when *p* < 0.05.

A priori power analysis, based on recommendations for TMJ research [48], indicated that a sample size of 300 participants provides 90% power to detect medium effect sizes (d = 0.3) at α = 0.05. Effect sizes were interpreted using the following thresholds: 0.1 (small), 0.3 (medium), and 0.7 (large). For categorical data, the phi coefficient (φ) was applied with the same thresholds. Results were evaluated for both statistical (*p* < 0.05) and clinical significance (effect size ≥ 0.3).

## 3. Results

Of the 1465 subjects who underwent an exhaustive clinical examination, 337 met the eligibility criteria, but eleven eligible individuals did not provide consent to participate in this study. Therefore, the sample size of the present study consisted of 321 students, namely 117 females and 144 males, aged 24.2 ± 3.3 years. Fifty-six (56) participants were attending humanitarian faculties, whereas 265 science faculty.

### 3.1. Prevalence of TMD Signs and Symptoms

When performing clinical examination, the two examiners, with a near perfect agreement (Cohen’s kappa coefficient = 0.96), found that 14.0% (*n* = 45) of students had pain disorder symptoms, 28.0% (*n* = 90) had joint disorders signs, 23.4% (*n* = 75) had both TMD signs and symptoms, and 34.6 (*n* = 111) had neither TMD signs nor symptoms.

Table 2 summarizes the presence of TMD signs and symptoms within the sample population divided according to gender and area of this study.

When analyzing the associations between TMD signs and symptoms and gender, a positive association was found between female gender and the presence of pain disorder symptoms. The effect size, while statistically significant, was small (φ = 0.23), suggesting modest clinical relevance.

Notably, the ratio of the prevalence of pain disorder symptoms between genders was F/M = 1:0.4. Similarly, pain disorder symptoms were more common among students in science faculties (S) compared to those in humanistic ones (H), with a ratio of H/S = 1:2, though with similarly small effect size (φ = 0.20).

### 3.2. Assessment of Psycho-Emotional Factors and Association with TMD Signs and Symptoms

The median scores of PHQ-9, PHQ-15, PHQ-4, and GAD-7 were 2.00 (interquartile range IQR = 5.00), 2.00 (IQR = 5.00), 3.00 (IQR = 6.00), and 3.00 (IQR = 6.00), respectively, suggesting negligible severity of anxious mood, depressed mood, and somatic symptoms in the sampled population.

Psycho-emotional factors of the sample population according to the DC/TMD Axis II assessment instruments are reported in Table 3.

The median score of PHQ-9, PHQ-15, PHQ-4, and GAD-7 in the female group was significantly greater than the median score in the male group, suggesting a higher level of anxiety, depression, and risk for somatoform disorders among female students compared to male students. While these differences were statistically significant, only the PHQ-15 and PHQ-4 showed a clinically meaningful effect size. Instead, no differences in the median score of questionnaires assessing psycho-emotional factors were found between students of the humanitarian and science faculties.

When analyzing the association between TMD signs and symptoms and psycho-emotional factors among students, higher scores of PHQ-9, PHQ-15, PHQ-4, and GAD-7 were found among students who reported pain disorder symptoms compared to those without pain (Table 4). According to TMJ research thresholds, these differences fell within the small-to-medium range of clinical relevance (d ≈ 0.3), with the PHQ-15 demonstrating the most consistent medium effect. Conversely, no differences in psycho-emotional factors were found between students with and without joint disorder signs (Table 4).

### 3.3. Assessment of Waking-State OBs and Association with TMD Signs and Symptoms and with Psycho-Emotional Factors

The assessment of waking-state OBs found a high presence of parafunctional behaviors in the sampled population (median = 20.0, IQR = 14.0). Notably, OBs were more frequent among female students (median = 21.0, IQR = 18.0) than male students (median = 18.0, IQR = 12.3; *p*-value < 0.001), and among students in science faculties (median = 20.0, IQR = 13.0) than students in humanitarian faculties (median = 15.0, IQR = 14.5, *p*-value = 0.011).

Subjects with pain disorder symptoms had a higher presence of OBs (median = 23.0; IQR = 17.0) compared to those without symptoms (median = 16.0; IQR = 14.0; *p*-value < 0.001). Instead, no differences were found in OBs’ presence between subjects with (median = 21.0; IQR = 16.0) and without (median = 19.0; IQR = 10.5) joint disorder signs (*p*-value = 0.167).

When analyzing the association between OBC score and the scores of the other DC/TMD Axis II assessment instrument, positive correlations were found between the presence of OBs and levels of anxious mood (*p*-value < 0.001), depressed mood (*p*-value = 0.001), psychological distress (*p*-value < 0.001), and risk for somatoform disorders (*p*-value < 0.001).

## 4. Discussion

The aim of the present cross-sectional study was to assess the prevalence of signs and symptoms of TMDs and OBs among university students, and to analyze their possible association with psychological and emotional factors, such as anxiety or depressed mood, psychological distress, and somatization.

The results found that TMDs are very common among university students, since less than 35% of the sampled population was free from signs and symptoms. Notably, 14.0% of students had pain disorder symptoms, 28.0% had joint disorders signs, and 23.4% had TMD signs and symptoms.

The prevalence of pain disorder symptoms was 37.3%, which is significantly higher than the prevalence assessed by Iodice et al. [1] in a previous study among adult Italian population samples, and by international surveys reporting prevalence in the general population ranging from 13% to 21% [49,50]. This discrepancy could be ascribed to several factors. First, the present study has been carried out after the COVID-19 pandemic, which may have led to an increase in TMD prevalence [51,52]. Secondly, the prevalence of TMD-related pain among students was found to be typically higher than that in the general population, as evidenced by previous investigations conducted among students from Jordan, Brazil, and Saudi Arabia [26,27,28]. The observed discrepancy between students and the general population may be attributed to the heightened anxiety and stress load characteristic of university life [48]. Such chronic stressors could dysregulate neurotransmitter systems (e.g., serotonin and dopamine), alter receptor expression patterns, and modify molecular pathways within key pain-processing brain regions, ultimately modulating pain perception thresholds and increasing susceptibility to musculoskeletal pain conditions [15,16].

A significant association was found between gender and pain disorder symptoms, since women were found to be 2.5 times more likely to develop them than men (Table 2). This data confirmed what was previously found by several epidemiological investigations, and could be attributable to biological, behavioral, psychological, and social differences existing between the genders [53]. Consistently, the overall level of anxious mood, depressed mood, psychological distress, and risk of somatization were found to be higher in women than men (Table 3).

Conversely, no differences in the prevalence of TMD signs were found between subjects divided according to gender, suggesting the possibly overwhelming role of the psychological sphere in pain disorders more than in disorders of function. Indeed, when assessing the association between TMD signs and symptoms and psycho-emotional factors among students, higher scores on the questionnaires of DC/TMD Axis II were found in subjects with pain disorder symptoms compared to asymptomatic ones, but no differences existed between students with and without joint disorder signs (Table 4).

Interestingly, students of science faculties were found to be more likely to suffer from TMJ and muscular pain than students of humanitarian faculties (Table 2). Similar results were previously found among Arabian and Jordanian students [26,27,28], and the authors assumed that the reason behind this could be the greater study load, competitiveness of science faculties compared to humanitarian ones, that led to higher levels of anxiety and stress. However, the present study did not find any differences between students of different faculties when assessing psycho-emotional factors (Table 3). This non-difference could be due to discipline-specific physiological awareness: the training of science students may increase their sensitivity in recognizing and reporting somatic symptoms, and their familiarity with biomechanical concepts may lower their perceptual threshold for musculoskeletal discomfort [54].

The assessment of waking-state OBs found a significant correlation between PHQ-9, PHQ-15, PHQ-4, GAD-7 scores, and OBC scores, suggesting that the presence of parafunctional behaviors is conceivably related to psycho-emotional factors. Indeed, female students, who were found to be psychologically more vulnerable, had higher OBC scores than men. Similarly, subjects with pain disorder symptoms had a higher presence of OBs than those without symptoms.

The above supports a possible chain of relationships between psycho-emotional factors, waking-state OBs, and muscular and skeletal pain, according to the following paradigm: anxious and depressed mood, psychological distress, and somatization may directly contribute to increase hypervigilance and amplify somatosensory pain; pain may result in maladaptive behavior such as parafunction; and parafunction may be a coping response to potential threat coupled with hypervigilance and somatosensory amplification [41,55].

Overall, the present study found a high prevalence of TMD signs and symptoms, and OBs among university students, significantly affected by a psychological background characterized by an even slight level of anxious mood, depressed mood, or stress.

The results of the present study are generalizable within the following limitations.

First, the cross-sectional design of this study restricts the ability to infer a causal relationship between variables. Secondly, a potential selection bias of participants should be considered since the sample was selected among students seeking dental examination, and they may not be representative of the general student population. Additionally, although a dental examination can also be aimed at prevention, most people request it because they present a symptom, which inherently biases the sample towards potentially symptomatic individuals. Secondly, the use of the OBC in the assessment of waking-state OBs should be considered as a possible limitation, since the clinical validity of the OBC has not yet been confirmed in the natural environments of participants. Lastly, some confounders were not considered, such as genetics, cultural background, and environmental factors.

Another study limitation involves the absence of radiographic imaging (e.g., MRI) for TMD diagnosis. Although advanced imaging could potentially enhance diagnostic precision, its systematic implementation in the student sample remains economically unfeasible and not essential for all patients. Indeed, the current methodology relies on DC/TMD clinical examination protocols that demonstrate excellent diagnostic sensitivity and specificity for the TMD disorders examined in this study.

However, the use of a standardized and validated diagnostic tool monitored TMD signs and symptoms, and the self-administration of questionnaires that enable responses that truly reflect the psycho-emotionality of respondents strengthens the external validity of the present findings.

Further investigations, including a control group of non-university students of the same age, are needed to confirm the present results. Moreover, a longitudinal study monitoring the presence of TMD signs and symptoms, OBs, and psycho-emotional factors before college enrollment, during the course of this study, and after graduation could be useful in determining whether college studies may be a risk factor for the onset of TMD.

## 5. Conclusions

The results of this study showed a statistically significantly higher prevalence of TMD signs and symptoms among university students compared to the general population and found a leading role of emotional and psychological factors in their etiology. Notably, significantly more pain-related symptoms were reported by female students than by male students, and a higher prevalence was shown by science faculty students than by humanitarian ones. However, the small effect sizes observed suggest that these differences, while consistent, may have limited generalizability. Moreover, maladaptive behaviors were demonstrated to be common coping responses to psychological distress.

This highlights the need for regular examinations for university students in order to allow early diagnosis and prompt management of TMDs, as well as psychological support and educational interventions to develop effective and healthy coping strategies to manage psychological threats.

## Figures and Tables

**Table 1:** Inclusion and exclusion criteria.

Inclusion Criteria	Exclusion Criteria
Undergraduate students registered in a degree program at the University of Salerno with no gender, age, or race restrictions.	Exchange students attending the Italian university for a limited time period. Poor knowledge of the Italian language. Medical or psychological conditions hindering patients’ comprehension or questionnaire completion (severe uncorrected blindness or low vision, low-functioning autism, dyslexia, dementia, severe post-stroke or post-traumatic cognitive disorders, moderate or severe intellectual disability, acute psychotic disorders, severe mood disorders with psychotic component or cognitive disorganization, and advanced multiple sclerosis with cognitive impairment). Identifiable orofacial pain causes other than muscle and temporomandibular joint pain (trigeminal neuralgia, neuropathic pain, burning mouth syndrome, migraine, cervical pain, atypical facial pain, fibromyalgia, dental pain, or atypical odontalgia). Missing data or adjustment for multiple tests in questionnaires.

**Table 2:** Prevalence of pain disorder symptoms and joint disorder signs among students divided according to gender and area of this study. Between-group differences were measured using the chi-square test.

		Gender					Area of Studies				
		F	M	*p*-Value	OR (CI 95%)	ES	H	S	*p*-Value	OR (CI 95%)	ES
Pain disorder symptoms	Yes	84	36	<0.001 *	0.369 * (0.23; 0.60)	0.23	14	106	0.035 *	2.00 * (1.04; 3.84)	0.20
	No	93	108				42	159			
Joint disorders signs	Yes	87	78	0.371	1.22 (0.79; 1.90)	-	23	142	0.089	1.66 (0.92; 2.97)	-
	No	90	66				33	123			

Symbols: “*”, statistically significant; Abbreviations: “ES”, effect size; “F”, female; “M”, male; “OR”, odds ratio; “H”, humanitarian faculties; “S”, science faculties.

**Table 3:** Assessment of psycho-emotional factors (PHQ-9, PHQ-15, PHQ-4, and GAD-7) of the students divided according to gender and area of this study according to the DC/TMD Axis II assessment instruments. Between-group differences were measured using the Mann–Whitney U test.

	Gender (Median, IQR)				Area of Studies (Median, IQR)			
	F	M	*p*-Value	ES	H	S	*p*-Value	ES
**PHQ-9**	3.00 (4.00)	2.00 (4.00)	0.030 *	0.18	3.00 (6.00)	2.00 (4.00)	0.341	-
**PHQ-15**	4.00 (6.00)	2.00 (2.00)	<0.001 *	0.29	3.00 (5.25)	2.00 (5.00)	0.957	-
**PHQ-4**	3.00 (4.00)	2.50 (4.00)	<0.001 *	0.27	3.00 (3.00)	3.00 (5.00)	0.378	-
**GAD-7**	4.00 (6.00)	2.00 (4.25)	0.003 *	0.20	3.00 (6.00)	3.00 (5.00)	0.295	-

Symbols: “*”, statistically significant; Abbreviations: “ES”, effect size, “IQR”, interquartile range; “F”, female; “M”, male; “H”, humanitarian faculties; “S”, science faculties.

**Table 4:** Association between TMD signs and symptoms and psycho-emotional factors (PHQ-9, PHQ-15, PHQ-4, and GAD-7) among the students. Between-group differences were measured using the Mann–Whitney U test.

	Pain Disorder Symptoms (Median, IQR)				Joint Disorder Signs (Median, IQR)			
	Yes	No	*p*-Value	ES	Yes	No	*p*-Value	ES
**PHQ-9**	3.00 (4.25)	2.00 (3.00)	<0.001 *	0.30	2.00 (6.00)	2.00 (4.00)	0.818	-
**PHQ-15**	5.00 (7.25)	2.00 (3.00)	<0.001 *	0.31	3.00 (6.00)	2.00 (5.00)	0.223	-
**PHQ-4**	4.00 (4.25)	2 (4.00)	0.002 *	0.27	3.00 (5.00)	3.00 (3.00)	0.195	-
**GAD-7**	4.00 (4.25)	2.00 (6.00)	0.001 *	0.28	3.00 (5.00)	3.00 (6.00)	0.961	-

Symbols: “*”, statistically significant; Abbreviations: “ES”, effect size; “IQR”, interquartile range.

## Data Availability

The datasets used and/or analyzed during the current study are available from the corresponding author upon reasonable request.

## Abbreviations

The following abbreviations are used in this manuscript:

DC/TMD	Diagnostic Criteria of Temporomandibular Disorder
GAD	Generalized Anxiety Disorder
H	Students of Humanitarian Faculties
IQR	Inter Quartile Range
OB	Oral Behavior
OBC	Oral Behavior Checklist
PHQ	Physical Health Questionnaire
S	Students of Science Faculties
TMD	Temporomandibular Disorder
TMJ	Temporomandibular Joint
WHO	World Health Organization
